# Critical Issues and Imminent Challenges in the Use of sEMG in Return-To-Work Rehabilitation of Patients Affected by Neurological Disorders in the Epoch of Human–Robot Collaborative Technologies

**DOI:** 10.3389/fneur.2020.572069

**Published:** 2020-12-22

**Authors:** Alberto Ranavolo, Mariano Serrao, Francesco Draicchio

**Affiliations:** ^1^Department of Occupational and Environmental Medicine, Epidemiology and Hygiene, INAIL, Rome, Italy; ^2^Department of Medical and Surgical Sciences and Biotechnologies, Sapienza University of Rome, Rome, Italy; ^3^Movement Analysis LAB, Policlinico Italia, Rome, Italy

**Keywords:** return-to-work rehabilitation, surface electromyography, instrumental-based biomechanical risk assessment, exoskeletons control, manual material handling monitoring

## Abstract

Patients affected by neurological pathologies with motor disorders when they are of working age have to cope with problems related to employability, difficulties in working, and premature work interruption. It has been demonstrated that suitable job accommodation plans play a beneficial role in the overall quality of life of pathological subjects. A well-designed return-to-work program should consider several recent innovations in the clinical and ergonomic fields. One of the instrument-based methods used to monitor the effectiveness of ergonomic interventions is surface electromyography (sEMG), a multi-channel, non-invasive, wireless, wearable tool, which allows in-depth analysis of motor coordination mechanisms. Although the scientific literature in this field is extensive, its use remains significantly underexploited and the state-of-the-art technology lags expectations. This is mainly attributable to technical and methodological (electrode-skin impedance, noise, electrode location, size, configuration and distance, presence of crosstalk signals, comfort issues, selection of appropriate sensor setup, sEMG amplitude normalization, definition of correct sEMG-related outcomes and normative data) and cultural limitations. The technical *and methodological* problems are being resolved *or* minimized also thanks to the possibility of using reference books and tutorials. Cultural limitations are identified in the traditional use of qualitative approaches at the expense of quantitative measurement-based monitoring methods to design and assess ergonomic interventions and train operators. To bridge the gap between the return-to-work rehabilitation and other disciplines, several teaching courses, accompanied by further electrodes and instrumentations development, should be designed at all Bachelor, Master and PhD of Science levels to enhance the best skills available among physiotherapists, *occupational health and safety technicians* and ergonomists.

## Introduction

Bipolar and multi-channel (high-density) surface electromyography (sEMG and HDsEMG) represent non-invasive physiological approaches, which enable greater comprehension of the upper limb, lower limb, and trunk muscle behaviors during the execution of movement (1–4). Differential bipolar sEMG signals, currently acquired using miniaturized wireless sensors attached to the skin, represent the spatio-temporal summation of all the motor unit action potentials, which propagate from the innervation zones to the tendon regions along the muscle fibers closest to the skin. In view of the complexity of these interference signals, simple indices facilitate an appropriate and complete analysis of the electrical activity of muscles. Simple indices provide information on “when” and “how much” the muscles are electrically active during the execution of both isometric and dynamic activities, and include the following parameters:

- amplitude indices (5, 6)
  ° maximum,
  ° averaged rectified value,
  ° root mean square, and
- muscle activation timings (7).

Furthermore, sEMG-based algorithms enable greater in-depth comprehension of the muscle coordination mechanisms adopted by the central nervous system (CNS) by estimating the following:

- simultaneous activation of several muscles or muscle groups (co-activation), a mechanism adopted by the CNS to stabilize joints, upper and lower limbs, and the spine (8–11),
- myoelectric manifestation of muscle fatigue, estimated by measuring the decrease in fiber conduction velocity (12, 13), which is reflected in an amplitude increase and spectral compression over time (14–16), and
- locomotor coordination (17–26), analyzed to comprehend how CNS lesions of neurological subjects with motor deficits influence plasticity and modular control of muscle patterns (27). It has been demonstrated that the CNS can linearly combine, with different weights, a limited number of basic functions called primitives or muscle synergies, to implement several motor tasks. During steady-state walking and running, five and four primitives, respectively, account for muscle activity (28–30).

High-density sEMG recordings are performed using high-density surface grids placed on the skin to evaluate the online spatial distribution of the sEMG activity and estimate the discharge times of several motor units by using decomposition algorithms (2, 31).

Despite the many advantages of sEMG and HDsEMG, these instrumental tools are largely underexploited and their application lags expectations in the fields of ergonomics and occupational medicine. These tools have begun to be applied in the prevention of work-related musculoskeletal disorders, a set of painful inflammatory and degenerative conditions affecting the joints, spinal dizcs, cartilage, muscles, tendons, ligaments and peripheral nerves, caused by manual lifting, pushing and pulling, repetitive movements, and patients handling activities (32–39). However, they remain underused in return-to-work rehabilitation plans for people with neurological pathologies with motor disorders.

**Figure 1:**
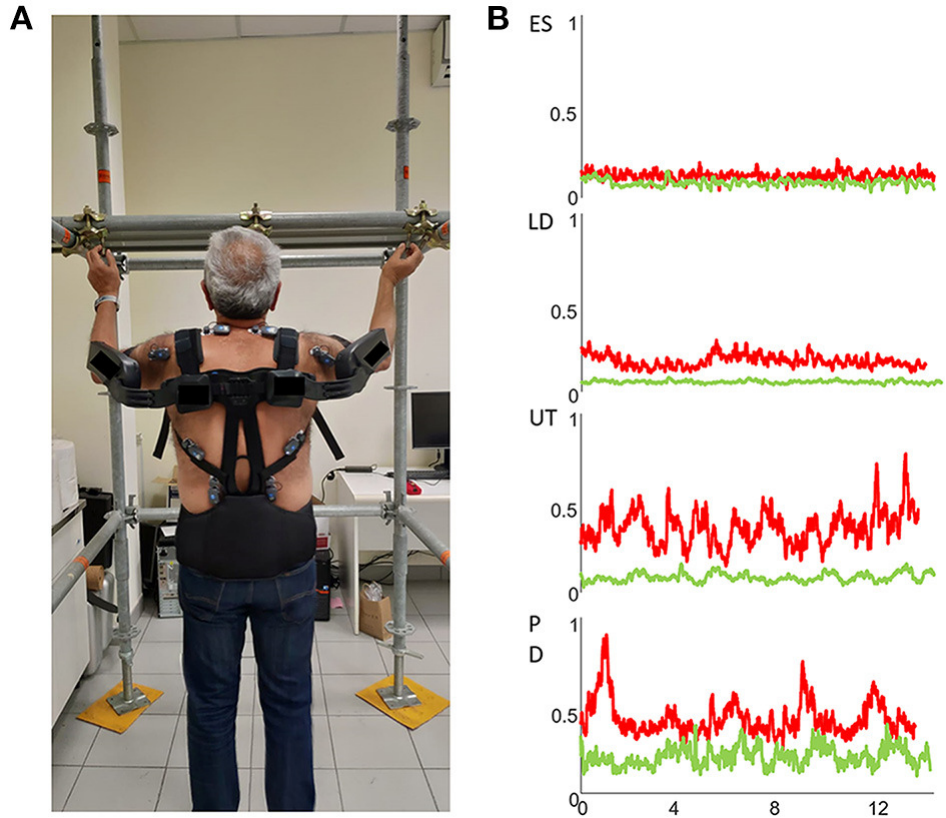
**(A)** Wearable wireless sEMG sensors placed bilaterally over the erector spinae (ES), latissimus dorsi (LD), upper trapezius (UT) and posterior deltoids (PD) muscles to assess the efficacy of a passive exoskeleton during the execution of an overhead screwing activity. **(B)** sEMG envelopes of right ES, LD, UT and PD muscles of a representative subject without (red traces) and with (green traces) the use of a passive exoskeleton.

People affected by neurological pathologies need to be integrated/reintegrated into their workplaces because their motor disease symptoms appeared when they were of working age, reducing their working capacity (40, 41) and employability (42). Recent studies have proven that avoiding early exit from employment plays a beneficial and key role in the overall quality of life of people affected by neurological disorders (41, 42). Clinicians manage their patients' premature work interruption (43, 44) by designing appropriate traditional and innovative pharmacological, surgical, and rehabilitation treatments, such as robotic rehabilitation, virtual reality, and neuromodulation (45–49). Furthermore, job accommodation plans are being enriched with new ergonomic options, such as work task rehabilitation and workplace interventions (50–52). In fact, the fourth industrial revolution has recently opened new occupational scenarios within which key human-robot collaborative (HRC) technologies, such as exoskeletons (Figure 1) and cobots, assist workers in their workplaces. Cobots can be defined as reconfigurable collaborative robots able to interact with workers within a shared space and to respond to the worker intentions and task variations in a timely manner while simultaneously offloading them from external loadings, and keep them in task-optimum and ergonomic working conditions. Small-medium enterprises can currently use collaborative technologies allowing flexible and ergonomic workplaces, which can adapt to the characteristics of workers with neurological disorders (53). Quite recently, the European Union's Horizon 2020 research and innovation program funded the SOPHIA (Socio-physical Interaction Skills for Cooperative Human-Robot Systems in Agile Production) project to establish and achieve, among others, the goal of validating the HRC technologies developed under the aegis of the project in the healthcare sector and in return-to-work rehabilitation of patients affected by neurological disorders. In particular, the European consortium is developing myoelectric HRC interfaces to study how new hybrid work environments can flexibly adapt to the human physical states and needs, thereby contributing to improvements in ergonomic interventions (53). Furthermore, the project has the aims, for prevention, to design training plans of professionals specialized in the workers motor performance measurement by using sEMG-based approaches, develop miniaturized wearable devices to monitor human-motor variables and render haptic stimuli to specific areas of the worker's body and develop new standards for adaptation of work environments and biomechanical risk assessment in collaborative manufacturing scenarios. Within this context, it is quite evident that sEMG can play a crucial role in complex vocational reintegration programs in classifying residual motor functions, assessing pre-post-rehabilitation and ergonomic interventions, and controlling wearable robotics. The professionals trained for the sEMG in return-to-work programs should be physiotherapists, *occupational health and safety technicians* and ergonomists which should operate in a multidisciplinary team also constituted by neurologists, occupational physicians, physiatrists, biomedical engineers and movement scientists. We believe that no new professions should be created.

*Occupational health and safety technicians* have an academic degree offered at Bachelor of Science level in the same field of physiotherapists (health professions sciences). Instead, professionals from different disciplines, for example occupational physicians, who aspire to practice the profession of ergonomist, need a degree that is not conferred by universities. In Europe, the Center for the Registration of the European Ergonomists (CREE) is the professional certification supported by European ergonomics associations and recognized by the International Ergonomics Association (IEA) which provides the title of “Eur.Erg.” and allows to practice the profession of ergonomist in 47 countries.

The above premise serves as a rationale for identifying and discussing the barriers to the coherent and widespread use of sEMG in work integration/reintegration.

This article represents the perspective, under the aegis of the SOPHIA project, of the Laboratory of Ergonomics and Physiology of the Italian Institute for Insurance against Accidents at Work (INAIL), a public non-profit entity aimed at facilitating the return-to-work of people with motor disorders.

## An Overview of sEMG Use in Return-To-Work of Patients With Neurological Disorders

Ethical review and approval and written informed consent were not required for the study because no human participants were recruited in the study and in accordance with the local legislation and institutional requirements.

Degenerative and acquired neurological diseases, including neuropathies, multiple sclerosis, stroke, spastic paraplegia, cerebellar ataxia, dystonia, traumatic spine and brain lesions, and encephalitis, are disorders, which can affect the motor function during working age and severely limit the autonomy and efficiency of workers (42, 54–58). The motor impairment of workers affected by neurological diseases may encompass several motor domains, including hand function, balance, and locomotion, resulting in considerable onus on the society in terms of reduced work productivity and cost. The main purpose of pharmacological, surgical, and rehabilitation treatments must be to improve the motor performance, autonomy, and daily lives of patients, thereby offering them the possibility of returning to work and optimizing their work capability.

Combined with kinematic and kinetic measurements, sEMG is currently widely used in research laboratories by movement scientists and still little in clinical routine by health operators to classify quantitatively the nature and degree of motor dysfunction, analyze the complex relationship between the primary deficit and the adaptive and compensatory mechanisms, categorize patients based on their specific neurological disease, and finally monitor pre-post-treatment. Importantly, as sEMG essentially investigates the final output of motor commands, it can quantify the residual motor function, which can theoretically be monitored continuously in workplace adaptation and integrated into HRC technologies.

### Application of sEMG in Monitoring

When a worker affected by a neurological pathology with motor disorders is reintegrated at work, an exhaustive assessment of his/her residual motor function is of primary importance to design and/or adapt his/her workplace well. Additionally, it is necessary to verify and monitor the efficacy of these ergonomic interventions over time.

Although no consistent studies have specifically investigated the sEMG of patients with neurological disorders in the workplace context, there are several reports on the residual muscle function assessment of patients with neurological disorders [(10), for review see (59, 60)]. Many muscle activation measures and indices exploring several aspects of motor control have been proposed for patients with neurological diseases: co-contraction/co-activation of single-joint or multi-joint muscles [(9, 23, 61), for review see (62)], spatio-temporal modular muscle activation (19–22, 27), muscle activation asymmetry (63), time-frequency coherences between joint muscle signals during dynamic contractions to detect the spasticity in the upper limbs (64), and muscle fatigue (65, 66). Furthermore, it is possible to obtain from these studies a set of functional measures to consider for work reintegration while simultaneously taking into account the uniqueness of the motor deficit specific to each disease.

Some long-lasting degenerative neurological diseases, such as cerebellar ataxia and spastic paraplegia, often begin at a young age and can persist for the entire duration of a subject's working life. This is of interest in the context of work-related rehabilitation because the majority of patients consider themselves capable of working, and approximately 78% of non-working patients seek employment (42). Furthermore, workers with cerebellar ataxia show low or average-to-low job stress-related risk (42).

The use of sEMG analysis in patients with degenerative cerebellar ataxia (for instance, spinocerebellar ataxia) reveals a series of muscle activation abnormalities (67). Specifically, patients with spinocerebellar ataxia show increased amplitude and duration of sEMG bursts in both the upper and lower (21, 22) limbs, with significant differences in muscle activation timing (21, 22). Further evidence reveals increases in both single-joint antagonist muscle co-activation (61) and multi-joint multi-muscle co-activation (68). All these abnormalities are related to the severity of the disease and balance features, suggesting they could be exploited as potential biomarkers for the work-related biomechanical risk evaluation and workplace adaptation monitoring of these patients. One possible approach is the planning of assistive devices for workplace adaptation, such as the use of supportive elastic suits (69). These soft wearable devices can improve movement stability and reduce the need to co-activate muscles. Consequently, they can lower the associated energy costs and tissue-overuse injuries owing to excessive compression and shear forces at, for instance, the L5-S1 joint of the spine.

It has been reported that patients with degenerative spastic paraplegia (for instance, hereditary paraplegia) show increased global (23) and segmental lower limb muscle co-activation (9), which correlate positively with disease severity, degree of spasticity, and energetic costs (9). In addition, when mapping the simultaneous activities of a large number of muscles during walking onto the anatomical rostrocaudal location of the motor neuron pools (70), patients with spastic paraplegia show an abnormal spread of muscle activation during gait, initially involving the sacral segments and, at more severe stages, the lumbar segments (20).

### Application of sEMG in Controlling HRC Technologies

Human-robot collaborative technologies, particularly exoskeletons, have proven significantly useful in the rehabilitation programs of patients affected by neurological diseases. Physiological parameters with sEMG play a key role in monitoring muscle activation amplitude and fatigue in the design of innovative active exoskeleton controller systems and assessment of their effectiveness on motor performance (71).

Recent improvements in the measurement and real-time classification of myoelectric signals have already facilitated the use of sEMG in man-machine interfaces for controlling prostheses and orthoses among other devices (72–76).

Very recently, human-exoskeleton interfaces were developed for the purpose of rehabilitation to support physically weak and disabled people in performing several motor activities of daily living, such as walking. These interfaces were developed based on new technical and mathematical approaches, including new sEMG signal-processing procedures (77–82).

The performance of these man-machine interfaces is quickly improving owing to neuro-musculoskeletal models driven by neural information obtained from the decomposition of HDsEMG (72).

## Barriers to sEMG Use in Return-To-Work of Patients With Neurological Diseases

The critical issues hindering the widespread adoption of sEMG in return-to-work programs are mainly attributable to technical, *methodological* and cultural limitations. The technical issues are attributable to both monitoring and control functions. With regard to monitoring, the most critical *technical* aspects are strongly associated with the sEMG technique:

- electrode-skin impedance, noise, and electrode contact stability;
- *while the methodological aspects are associated to:*
- problems linked to electrode location, size, configuration, and distance;
- presence of crosstalk signals (16).
- placement of sEMG electrodes for long hours;
- selection of the right sensor setup on the base of the neurological pathology and manual handling activity to be investigated;
- management the sEMG amplitude normalization;
- definition of appropriate sEMG-related outcomes and normative data.

Fortunately, the effect of these critical issues on the sEMG signal quality can be reduced with the aid of authoritative reference books and tutorials. In particular, the European Recommendations for Surface Electromyography (83), which needs to be updated, and the Atlas of Muscle Innervation Zones (84) are recommended as guides for the use of sEMG together with recent tutorials and consensus papers (85–87). A knowledge of the contents of these texts and tutorials makes users aware of the current limitations of the sEMG approach given its ability to monitor only a limited number of superficial muscles. Despite this, the technical *and methodological* limitations of the sEMG approach can be *minimized* and do not justify its non-usage in return-to-work rehabilitation plans. In addition, new wearable sensors and electronic smart devices such as smartphones and tablets allow simple monitoring of the worker at the workplace. Wearable sensors do not interfere with the typical movements performed by workers owing to their miniaturization and wireless communication protocols. In addition, multi-channel sEMG systems are available in wireless versions despite their high number of channels and high data rate. The combination of sensor networks and intelligent work environments provides real-time estimation of physiological parameters, enabling direct feedback to workers who are monitored directly and constantly at the workplace. Real-time monitoring is additionally useful for providing acoustic, visual, and vibro-tactile stimuli to workers (53, 88) executing manual handling tasks in awkward postures or requiring significant physical effort, or when muscle fatigue sets in.

As regard the sEMG use in work environment, each manual handling activity represent a risk of onset of well-classified diseases affecting the musculoskeletal system. For instance, lifting activities imply high compression and shear forces at L5-S1 joints (strongly correlated with muscle co-activation) with a significant involvement of erector spinae and rectus abdominus muscles, while handling low loads at high frequency imply neck and upper limbs muscles fatigue. For this reason the choice of channels and the rationale for selecting them should be task-guided.

The criticisms related to the use of sEMG to control collaborative wearable trunk and upper limb devices designed to assist people with neurological disabilities are attributable to the algorithms used in human-robot interfaces. These algorithms are used for pattern recognition and classification of patients' movement intentions. Only a few years ago, the performance of sEMG-based interfaces had not reached accuracies acceptable for widespread commercial use (74). Accuracy was limited by the high inter-subject variability, which required subjective calibrations and training. Fortunately, these interfaces, including machine-learning algorithms, have since been significantly improved, enabling the acceptable optimization of HRC control mostly for people with severe upper and lower limb disabilities (89).

Another technical limitation of sEMG-based interfaces for use in HRC technology control is the fact that most wearable assistive devices use traditional control tools, such as bipolar sEMG, to record antagonist muscle activities. This low spatial sampling implies that a maximum of one degree of freedom (DoF) can be controlled. Furthermore, managing up to two DoFs requires slow, sequential, and unintuitive control. The related limited functionality in conjunction with the extensive training required of neurological subjects led to the high rejection rates of these technologies (72). Currently, classification and regression approaches outperform traditional control tools in controlling complex motor activities in terms of speed and accuracy, providing a promising method for advanced myoelectric control (72).

Without doubt, in job integration/reintegration, the sEMG approach has yet to be adopted and the critical issues associated with it are managed with difficulty. In fact, while reasonable workplace accommodation and disability employment issues are being historically and widely addressed by the governments of the industrialized world, the adoption of instrument-based quantitative assessments of ergonomic interventions *has so far been disregarded*, owing to cultural barriers, which lead to a preference for qualitative approaches.

A clear testimony of the presence of this educational gap is the worldwide social policies for persons with disabilities (90, 91), such as the European Disability Strategy (2010–2020) and the directives 89/654/EEC, 2000/78/EC, and 2000/78/EC are the corresponding policies. Another example is that of the Job Accommodation Network, a facility of the United States Department of Labor's Office of Disability Employment Policy, which provides a valuable strategy for the inclusion of people with neurological disabilities (92). Nevertheless, only half of the population with disabilities has been accommodated well in terms of workplace design and there is evidence of poor knowledge about adaptation of workers with neurological pathologies.

The lack of quantitative approaches suggests that, except in rare cases, sEMG is not taught in the Ph.D. programs of universities and occupational medicine specialization schools. Furthermore, the domestic and international chapters of the Human Factors and Ergonomics Society do not actively promote meetings, conferences, and events to address the challenges of sEMG use. Finally, the reference society for sEMG, the International Society of Electrophysiology and Kinesiology, has thus far not dedicated specific training programs regarding job accommodation. All the above mentioned training activities would typically be successful to address methodological issues, as increased knowledge alone cannot overcome technical limitations.

The enormous potential of the sEMG and HDsEMG approaches and their very limited use in return-to-work programs represent a real paradox. It is difficult to determine why sEMG is underused. Perhaps, the most likely reason is that the training provided to professionals is based on more qualitative rather than quantitative approaches, and the transition from one approach to the other is evidently difficult. Professionals in the field have to be trained to understand the extremely variable abnormalities of workers suffering from neurological pathologies and to associate an appropriate return-to-work plan with them.

## Discussion

Although sEMG is considered the most informative instrument for muscle monitoring when wearable robots are used, its use poses a number of challenges.

One such challenge, which we believe is on the verge of being addressed, pertains to the use of exoskeletons for active rehabilitation therapies. It pertains to the optimization of appropriate smart algorithms to detect patients' intentions and allow exoskeletons to act in synergy with them (93, 94). The challenge is to enable symbiotic physical HRC by incorporating accurate subject-specific computer models of each individual's neuro-musculoskeletal system to enable appropriate anticipation mechanisms. This is crucial to estimate muscle and joint stiffness accurately to determine the onset of excessive rigidity, which may be related to fatigue or negative compensatory strategies (95).

Myocontrol should be increasingly based on HDsEMG to increase spatial resolution with respect to low-density sEMG and to improve the accuracy of motor workers' intentions recognition. Moreover, machine learning approaches such as artificial neural networks should be used to evaluate the capacity of workers with neurological diseases for myocontrol.

In a recent study (96), HDsEMG was used to test the ability of participants with Duchenne muscular dystrophy (DMD) to produce repeatable HDsEMG patterns, which were unexpectedly comparable with those of healthy participants, suggesting a clear potential for the myocontrol of wearable exoskeletons. High-density sEMG can be applied to analyze the altered motor control of people with DMD and potentially interface them with assistive wearable robots. In addition, non-invasive decoding of individual α-motor neuron activation may represent a new option for the design of real-time closed-loop control applications, such as transcutaneous and epidural electrical stimulations.

To bridge the gap between the return-to-work concept and other disciplines, several educational activities should be developed to enhance and apply the best skills available in rehabilitation engineering, physiotherapy, occupational therapy, and ergonomics. For a few years, INAIL has been promoting a Ph.D. program together with the Sapienza University of Roma titled “Kinematic, Kinetic, and Electromyographic Characterization of Motor Disabilities and Biomechanical Overload Risk Management for Job Reintegration,” a Masters' course titled “New Methodologies for the Evaluation and Management of Biomechanical Risk and Criteria and Methods for the Adaptation of Workplaces,” and several training courses regarding the role of sEMG in occupational medicine and ergonomics. The participation, although the events were not free and not recognized as continuing education, has always been conspicuous with a number of participants (*occupational health and safety technicians*, physiotherapists, ergonomists, occupational physicians, rehabilitation engineers and movement scientists) that has always reached the maximum allowed limit (30 in the case of training courses). The authors of this article begin to observe a first positive impact of these initiatives on the need that operators in the field have in using the sEMG approach.

These mandatory educational opportunities must capitalize on the skills of the leaders and innovators of sEMG to serve physiotherapists, health and safety technicians, and ergonomists by providing them with qualified training focused on the management of the monitoring and HRC technologies and instrumental recordings. In particular, these professionals should know the physiology of sEMG signals, electrodes placement, software and hardware for acquisition. Furthermore, they are expected to know basic and some more complex concepts of signal processing, do a general visual interpretation and be able to generate reports that can be interpreted by the other team members. The multidisciplinary team should plan, implement and evaluate the return-to-work program in both the clinical and occupational environment. It should be considered that when patients with neurological disorders are involved, the key professionals leading the team should be neurologists in the health sector and occupational physicians at workplace. INAIL is organized throughout the national territory in order to fully manage the job integration/reintegration process but this service is also offered by consultant companies. Rehabilitation engineers should be given greater options to work with patients and physiotherapists, health and safety technicians end ergonomists. We believe that no other hybrid professions should be created. The contents must be based first on elementary concepts [which are illustrated in free online materials, such as those available at [99]] and, second, on integrated approaches promoting the culture and acceptance of instrument-based quantitative methodologies. All these actions should be taken as early as possible by guiding workers with chronic neurological disorders to return to work and stay in work with well-managed occupational safety and health interventions. The abovementioned activities may additionally yield tangible savings for businesses and national health and welfare systems.

Obviously, the teaching activities directed to strategic professions alone cannot solve the problem of under-use of sEMG in return-to-work plans. In fact, they must be accompanied by further electrodes and instrumentations development especially from the perspective of emerging artificial intelligence that may be encapsulate sEMG knowledge. Such tools may provide the professional with information to act on, thereby reducing the current “art” aspect of using EMG to something more in everyone's hands to use and exploit.

In addition to the motor impairments, cognitive and speech impairments are major contributors that strongly impact on the returning to work. Important aspects to consider are to understand how patients with cognitive and speech problems can adapt to the sEMG monitoring and how they can be assisted by using specific sEMG technology (96).

There are other challenges that are specific to application of sEMG in return-to-work environment according to the specific motor impairment characterizing the different neurological diseases. For instance, muscle fatigue, which is a common feature of several neurological diseases (i.e., multiple sclerosis, stroke, muscle dystrophy) results in altered motor unit recruitment and decreased maximal voluntary motor unit firing rate that can be detected by sEMG monitoring. A specific scenario could be to adapt the workplace and to modify the work-task according to the subjects' abnormal fatiguing performance, by assisting the workers with devices and/or reducing the amount and the duration of the work-related activity. Furthermore, an individualized rehabilitative program could be planned to improve the impact of the work-task on the fatigability in a long-term period.

The abnormal muscle co-activation is another example of common problem identifiable by sEMG in several neurological disorders (e.g., cerebellar ataxia, Parkinson disease, multiple sclerosis, stroke) which can be detected by measuring the simultaneous time-varying sEMG signal in many muscles. It is known that patients with balance disorders increase the muscle co-activation to control their walking instability in the attempt to stiffen the body segments. Unfortunately, this compensatory mechanism has some negative effects, such as increased metabolic cost and risk of cartilage degeneration. A specific scenario for these workers is to plan an appropriate workplace rehabilitation to improve their balance, to stabilize the body segments and to reduce the need to increase the muscle co-activation.

In conclusion, sEMG should be used in job integration plans to:

- classify the nature and degree of residual motor function in order to design/adapt the workplace;
- assess the efficacy of work-task rehabilitation and ergonomic interventions;
- control new assistive technologies such as collaborative robots;
- evaluate the biomechanical risk during the execution of manual handling activities;
- plan a preventive rehabilitation program to prevent injury.

Including the sEMG approach in work integration/reintegration offers the possibility of designing programs based on the residual motor abilities of the worker and adapting his/her workplace. This allows to consider a wide range of workers with several neurological pathologies and different levels of severity but without a complete inability to perform activities of daily life.

To make this possible, Bachelor, Master and PhD programs should be promoted, or at least, supervised and monitored, by scientific societies in the fields of physiotherapy, ergonomics, occupational medicine, biomechanics, electrophysiology and kinesiology, and should include continuing education courses on the use of sEMG specifically oriented to teachers in these fields.

## Data Availability Statement

The raw data supporting the conclusions of this article will be made available by the authors, without undue reservation.

## Ethics Statement

Ethical review and approval and written informed consent were not required for the study as per local legislation and institutional requirements.

## Author Contributions

AR, MS, and FD planned the manuscript, performed the literature searches, wrote the text and revised the manuscript. All authors contributed to the article and approved the submitted version.

## Conflict of Interest

The authors declare that the research was conducted in the absence of any commercial or financial relationships that could be construed as a potential conflict of interest.
